# Correlation between pterygium and dry eye: diagnostics and risk factors not considered

**DOI:** 10.5935/0004-2749.2022-0240

**Published:** 2025-02-11

**Authors:** Camila A. Martinez Cornejo, Erick R. Moscoso Levano

**Affiliations:** 1 School of Human Medicine, San Juan Bautista Private University, 108 street Luis Albilla, as Viñas Urbanization (ex Toche), Chincha, Ica, Peru

Dear Editor,

In our article “Correlation between the presumed pterygium with dry eye and with systemic and ocular risk factors,” the assessment for the categorization of presumed pterygium in the participants was performed solely by the observation of a fibrovascular growth extending from the conjunctiva to the limbus and enveloping the cornea^(1)^. In the presence of pathologies with similar characteristics (pinguecula, pseudopterygium, spinocellular carcinoma, and nodular scleritis), it is necessary to perform additional tests, such as slitlamp examination, histopathology, and biopsy through optical coherence tomography, that allow differentiation between benign and malignant pathologies within the above-mentioned diagnoses^(2)^.

The omission of important risk factors in this study, such as exposure to ultraviolet radiation without adequate protection (UV filter)^(2)^, previous history of passive or active smoking^(3)^, the presence of environmental pollutants^(4)^, and the type of lenses in the case of ophthalmic frames, could affect the results^(5)^.

Studies have shown that prolonged exposure to solar radiation without the protection of lenses with UV filters is directly related to the development of the first^(2)^ and the second pathologies (pterygium and dry eye); the particles eliminated during tobacco and nicotine consumption produce inflammation of the ocular surface and affect the stability of the lipid layer of the tear film produced by the meibomian glands^(3)^, which could explain the high probability that smokers may develop or increase their probability of suffering from dry eye. Additionally, harmful agents, such as environmental pollutants, may affect at ocular level and increase the probability of suffering from these pathologies^(4)^. The use of ophthalmic frames with special lenses (blue-blocking) is relevant, since they protect the eye from blue light emitted by electronic devices, which are associated with these conditions when they are used for a long period^(5)^. This factor should be considered in people with visual acuity who wear glasses occasionally or permanently.

In conclusion, given the existence of different ocular pathologies with the same clinical pathological characteristics as pterygium, it is necessary to perform additional diagnostic procedures, as well as to include any other risk factors for both pathologies mentioned, because people are exposed to them daily.
